# Development of an Electronic Medical Record Based Alert for Risk of HIV Treatment Failure in a Low-Resource Setting

**DOI:** 10.1371/journal.pone.0112261

**Published:** 2014-11-12

**Authors:** Nancy Puttkammer, Steven Zeliadt, Jean Gabriel Balan, Janet Baseman, Rodney Destiné, Jean Wysler Domerçant, Garilus France, Nathaelf Hyppolite, Valérie Pelletier, Nernst Atwood Raphael, Kenneth Sherr, Krista Yuhas, Scott Barnhart

**Affiliations:** 1 International Training and Education Center for Health, Department of Global Health, University of Washington, Seattle, Washington, United States of America; 2 Department of Health Services, University of Washington, Seattle, Washington, United States of America; 3 International Training and Education Center for Health—Haiti, Port-au-Prince, Haiti; 4 Department of Epidemiology, University of Washington, Seattle, Washington, United States of America; 5 Division of Global HIV/AIDS, Centers for Disease Control and Prevention, Port-au-Prince, Haiti; 6 Population Division, Ministry of Public Health and Population, Port-au-Prince, Haiti; 7 Health Alliance International, Department of Global Health, University of Washington, Seattle, Washington, United States of America; 8 Center for AIDS Research, Department of Global Health, University of Washington, Seattle, Washington, United States of America; 9 Department of Medicine, University of Washington, Seattle, Washington, United States of America; University of Athens, Medical School, Greece

## Abstract

**Background:**

The adoption of electronic medical record systems in resource-limited settings can help clinicians monitor patients' adherence to HIV antiretroviral therapy (ART) and identify patients at risk of future ART failure, allowing resources to be targeted to those most at risk.

**Methods:**

Among adult patients enrolled on ART from 2005–2013 at two large, public-sector hospitals in Haiti, ART failure was assessed after 6–12 months on treatment, based on the World Health Organization's immunologic and clinical criteria. We identified models for predicting ART failure based on ART adherence measures and other patient characteristics. We assessed performance of candidate models using area under the receiver operating curve, and validated results using a randomly-split data sample. The selected prediction model was used to generate a risk score, and its ability to differentiate ART failure risk over a 42-month follow-up period was tested using stratified Kaplan Meier survival curves.

**Results:**

Among 923 patients with CD4 results available during the period 6–12 months after ART initiation, 196 (21.2%) met ART failure criteria. The pharmacy-based *proportion of days covered* (PDC) measure performed best among five possible ART adherence measures at predicting ART failure. Average PDC during the first 6 months on ART was 79.0% among cases of ART failure and 88.6% among cases of non-failure (p<0.01). When additional information including sex, baseline CD4, and duration of enrollment in HIV care prior to ART initiation were added to PDC, the risk score differentiated between those who did and did not meet failure criteria over 42 months following ART initiation.

**Conclusions:**

Pharmacy data are most useful for new ART adherence alerts within iSanté. Such alerts offer potential to help clinicians identify patients at high risk of ART failure so that they can be targeted with adherence support interventions, before ART failure occurs.

## Introduction

HIV antiretroviral therapy (ART) requires adherence levels of >90% in order to optimize therapeutic benefit [1], [2]. Patients with lesser adherence run the risk of incomplete HIV viral suppression and developing resistance to ART medications. In resource-limited settings, the high cost and limited availability of second-line ART regimens make it critical to minimize ART therapeutic failures with first-line regimens.

Early identification of gaps in ART adherence can help prevent ART failure. However, measuring ART adherence is a challenge [3]. Many of the techniques considered to be most valid, such as examination of drug concentrations in tissue samples, medication electronic monitoring system caps, and unannounced pill counts during home visits, are costly to implement and impractical in resource-limited settings [4], [5]. Self-reported measures gathered on a routine basis during clinic visits are more practical, but may be biased due to poor recall or social desirability [1], [6], [7], [8]. Pharmacy-based adherence measures derived from ART dispensing data can also be practical in resource-limited settings, depending on availability and quality of data [8], [9].

The adoption of electronic medical record systems in resource-limited settings can help clinicians monitor patients' ART adherence and identify patients at risk of future ART failure, allowing resources to be targeted to those most at risk. This study used data from the iSanté electronic medical record system in Haiti to develop and validate a prediction model for risk of ART failure, based upon self-reported and pharmacy-based adherence measures as well as other patient demographic and clinical data.

## Materials and Methods

### Study Setting and Patient Population

The study cohort included adult (age ≥15 years) HIV patients from Hôpital St. Michel in Jacmel (HSM) and Hôpital St. Antoine in Jérémie (HSA) who initiated a life-long ART regimen between January 1, 2005 – June 30, 2013. ART regimens used for prevention of mother-to-child HIV transmission (PMTCT) or other prophylaxis were not considered to be life-long regimens and were excluded from the analysis (although patients using ART for prophylaxis had the opportunity to enter the analysis later, upon initiating a life-long regimen).

Both hospitals provide primary and secondary levels of service and serve as the main referral hospitals in their respective departments, which are rural and mountainous regions of Haiti. Adult HIV prevalence is estimated to be 2.1% in the region served by HSM and 1.5% in the region served by HSA [10]. Both facilities began their ART programs in early 2005, and by mid-2013 had together enrolled 2,510 patients on ART.

### Data Source

All study data came from the consolidated server for the Haitian Ministry of Public Health and Population's (MSPP) iSanté electronic medical record (EMR) system. The iSanté system is used in more than 100 health care facilities and contains longitudinal health records on approximately 300,000 patients, including approximately half of the 42,000 Haitians receiving ART in 2012 [11], [12], [13].

iSanté was first deployed in Haiti in April 2005, based upon standardized HIV clinical care encounter forms, laboratory forms, and pharmacy forms originally validated and disseminated by the MSPP in October 2004. Over time, the MSPP added encounter forms for ancillary HIV services like ART adherence assessment, counseling, and home visits. At the facilities, use of the iSanté EMR system evolved from retrospective data entry of paper forms toward point-of-care use at both hospitals. The EMR interface closely matches the format and content of the paper forms, with an emphasis on structured data elements rather than free text. While the electronic system contains data validation rules and warnings, it places few hard constraints on data entry and has few mandatory data elements.

First use of iSanté began in March 2006 at HSA and April 2006 at HSM, and the system was used to retrospectively capture data for ART patients who initiated treatment as early as January 2005 at HSA and March 2005 at HSM. The ART adherence assessment form was added to the system in February 2008.

Data obtained from the consolidated data repository were de-identified and the study close date was June 30, 2013.

### ART Adherence Measures

We explored five types of adherence measures using iSanté data. The first three were pharmacy-based measures: 1) medication possession ratio (MPR), or total quantity of medication of any type dispensed divided by the number of days in the calendar period; 2) proportion of days covered (PDC), or the proportion of days during the period which are covered by the most recently prescribed medication (a more conservative estimate of adherence than MPR since it excludes left-over medication stockpiled from prior prescriptions); and 3) timely visit ratio (TVR), or the proportion of pharmacy refills collected no more than 2 days after the expected refill due date. Our procedures for calculating MPR and PDC used dispense dates and the number of days of medication dispensed, based on standard definitions [14], [15] and methods of calculation [16]. Since MPR can exceed 100% when patients pick up refills before exhausting earlier medication supplies, we truncated our measure of MPR measure at 100% to represent maximum possible adherence. The remaining two were self-reported measures collected by clinicians or pharmacists using the standard adherence assessment form during regular clinical visits. These were: 4) the average level of medications taken during the past 30 days, based on a visual analogue scale (VAS); and 5) the proportion of adherence assessments where patients reported no missed doses during the last 3 days (%NoMD). The VAS used a standard, validated instrument translated from English [17]. Baseline adherence measures during the first 6 months on ART were used in the analyses.

### Study Outcome

The outcome of interest was ART failure, based on World Health Organization (WHO) immunologic and clinical criteria. A patient was considered to have failed ART if they met any of the following criteria: 1) fall in CD4 cell count to the baseline level or below; 2) fall in CD4 to below 50% of the peak value following ART initiation; 3) persistent CD4 cell count of <100 cells/µl; or 4) onset of a new WHO stage IV diagnosis not reflective of immune reconstitution syndrome [18]. According to Haitian national treatment guidelines, presence of any of these criteria after 6 months of ART use can trigger consideration of a switch to a second-line ART regimen [19]. Clinical diagnosis data within iSanté were fairly incomplete, and could not be used in absence of CD4 data to differentiate ART failures from non-failures. We restricted the primary and secondary analyses to patients with both baseline and follow-up CD4 measures and allowed this group to meet the ART failure definition based on either WHO immunologic or clinical criteria.

**Figure 1:**
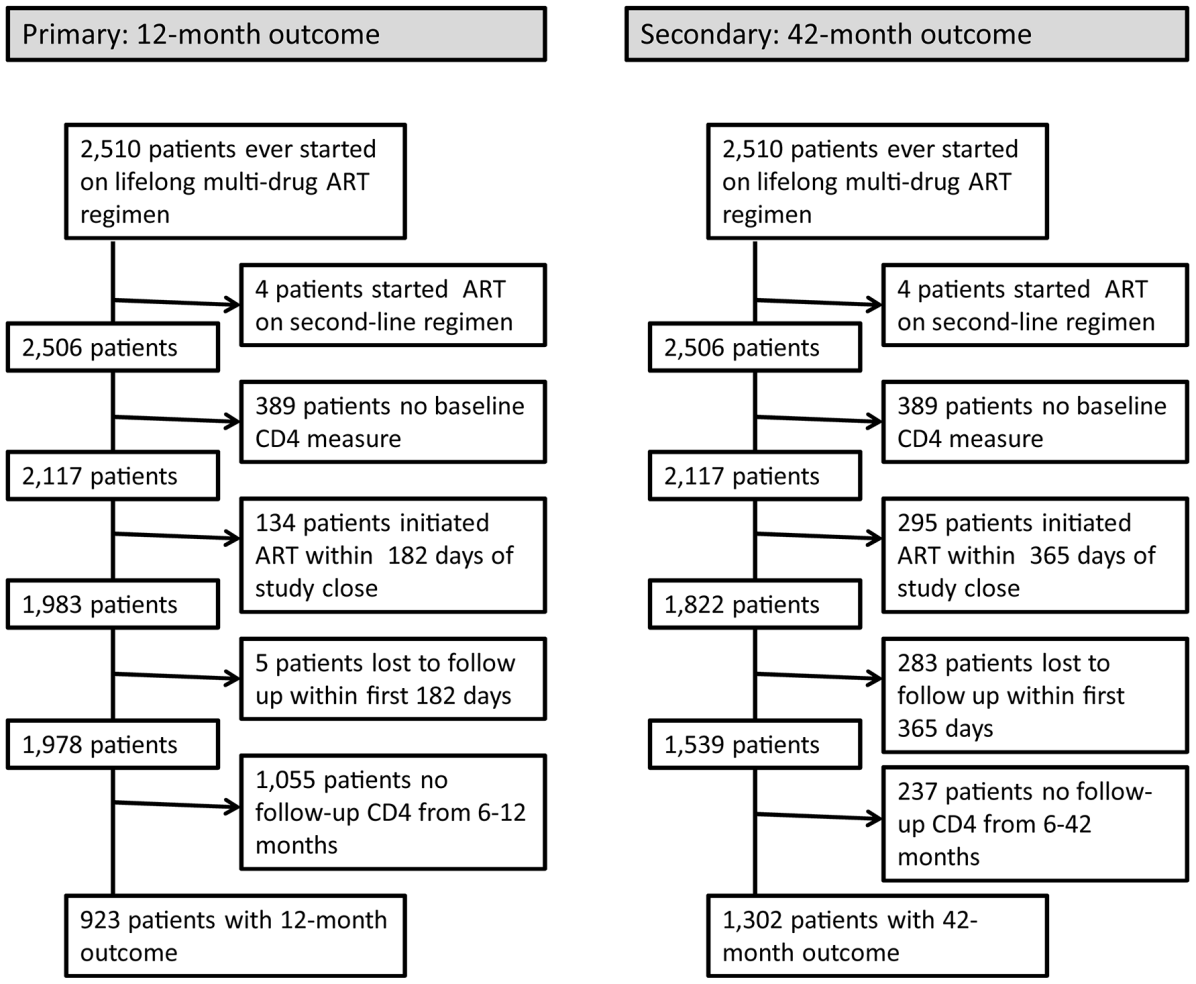
Flow Chart for Participants in the 12-Month and 42-Month Analyses.

Our primary analysis considered ART failure outcomes observed during months 7–12 following ART initiation, while our secondary analysis considered ART failure outcomes observed during months 7–42 following ART initiation (**Figure 1**). The baseline CD4 level was the measure occurring closest in time to the ART start date, during the period from 180 days before ART initiation to 7 days after ART initiation.

### Covariates

Covariates considered in the analysis were: hospital facility, gender, marital status, age at ART initiation, location of residence relative to the hospital, type of initial ART regimen, duration of enrollment in HIV care prior to ART initiation, year of ART initiation, baseline CD4 cell count, baseline body mass index (BMI), number of ART adherence assessments conducted during the first 6 months on treatment, and whether any ART adherence assessment was done by a clinician. We had data available on commune of residence, so location of residence was grouped by residence in: 1) the same commune as the hospital; 2) an adjacent commune bordering the commune of the hospital; or (3) a non-adjacent commune.

### Data Validation and Cleaning

We conducted a data validation exercise to assess the concordance between information in the EMR and information on paper-based forms at the two hospitals. Among a random sample of 200 ART patients, concordance of data exceeded 90% for date of ART enrollment and exceeded 95% for year of birth, gender, CD4 value, ART medication prescribed, and number of days of medication prescribed [20].

We applied a limited set of data cleaning rules to the electronic data, suitable for eventual use within an automated algorithm to be run in real-time against patient data within the live iSanté database. These rules were applied to patient registration dates, visit dates, medication dispense dates, patient BMI, and patient ages. The rules included logic for selecting dates when implausible date relationships existed (i.e. when a visit date fell before a registration date), excluded observations of weight or height which fell outside of humanly plausible bounds, and imputed patient ages when day or month of birth was missing (rules available upon request from the corresponding author).

The minimum quantity of medication dispensed for any single drug within a multi-drug regimen was used to calculate ART regimen refill due dates. For dispenses with missing quantities of medication dispensed, the between-visit time interval was excluded from both the numerator and denominator of the pharmacy-based adherence measures. Covariates with missing data were treated as categorical variables with a missing data category.

### Statistical methods

#### Descriptive statistics

We examined patient characteristics and baseline ART adherence measures among those with and without ART failure observed during the 7–12 months following ART initiation, and compared observed values between the two groups using Pearson's Chi-square test, Student's t-test, or the Wilcoxon rank sum test for continuous variables with skewed distributions. We also compared patient characteristics and baseline adherence among patients included vs. excluded from our analyses, to identify any selection issues.

#### Primary analysis, Step 1: Selection of adherence measure

First, we assessed how well each of the five baseline adherence measures predicted ART failure by 12 months. Patients retained within the 12-month analysis had at least 182 days of study follow-up after ART initiation and had a follow up CD4 measure taken from 182–365 days after ART initiation (n = 923, see **Figure 1**). Logistic regression models using continuous forms of each measure were used to test prognostic accuracy, using the area under the receiver operating characteristic curve (AUC) [21], [22]. The best performing measure was considered to be the measure which offered the highest AUC.

#### Primary analysis, Step 2: Selection of patient characteristics

Next, we considered enrichment of the prediction model using additional covariates beyond the selected adherence measure. Patients with 12-month outcomes were split randomly into 50∶50 derivation and validation sub-samples [23]. A forward stepwise selection procedure, using the Akaike Information Criterion (AIC), was used to identify a robust yet parsimonious prediction model in the derivation sub-sample [24]. The performance of the resulting prediction model was then assessed in the validation sub-sample. For continuous covariates retained in the model following the stepwise procedure, we categorized continuous covariates into deciles to identify the dichotomous level of the covariate at which predicted risk increased markedly, and then compared performance of continuous and binary forms of the covariates to assess whether performance degraded with simplified, binary versions of the covariates. We used dichotomous versions of each covariate in the final prediction model.

#### Primary analysis, Step 3: Formulation of a risk score and risk groupings

A risk score, taking into account the relative weight of each covariate, was constructed by multiplying the estimated coefficients from the logistic model by 10 and rounding to the nearest decimal place and then multiplying this quantity by the covariate value [25], [26], [27]. We assessed the performance and calibration of the risk score in the validation sample, using the AUC and the Hosmer-Lemeshow goodness of fit test [28]. Finally, we created a risk grouping with low, medium and high categories based on tertiles of the risk score distribution, and examined performance and calibration of the risk groupings using similar methods.

#### Secondary analysis: ART failure by 42 months

Our goal in this secondary analysis was to further validate the formulation of the risk score and risk groupings and to assess their performance in predicting ART failure over a longer time frame, through 42 months following ART initiation. For this analysis, we used time-to-event, or survival analysis, methods [29]. Patients retained within the 42-month analysis had at least 365 days of study follow-up after ART initiation (n = 1,302, see **Figure 1**). Each patient contributed up to seven 6-month periods to the analysis and time to first ART failure was the right-censored outcome. Patients were followed from the date of ART initiation until the period of ART failure, the period prior to the study close date, or the period where loss to follow-up occurred, whichever was first. Loss to follow-up was defined as having 180 days pass with no clinical encounter.

To compare the performance of the adherence measures in the survival analysis framework, we created standardized versions of the baseline adherence measures using z-scores. Then we assessed each measure's ability to predict ART failure in separate Cox regressions. The standardized adherence measure with the largest magnitude regression coefficient was taken to offer strongest prediction capability. Next, we applied the risk score and risk groupings derived in the primary analysis to the survival data. We stratified the Kaplan-Meier survival curves by risk groupings and used the log-rank test to assess differences in the survival curves across the groups over the extended 42-month time frame.

#### Sensitivity analyses

We explored the robustness of the results in the context of incomplete baseline CD4 cell count data. Specifically, we relaxed the requirement for a baseline CD4 measure to be available, assuming that these patients could still be observed to fail based on WHO criteria 2–4. All the steps in the primary and secondary analysis were repeated; however, baseline CD4 was modeled with a missing value category.

### Ethics Statement

The study protocol was reviewed by the University of Washington and received a human subjects exemption based upon use of existing de-identified patient data. It was also reviewed by the US Centers for Disease Control and Prevention and approved as a non-research program evaluation, and was reviewed and approved by the Haiti National Bioethics Committee.

## Results

### Analysis Cohorts and Availability of Adherence Data

There were 923 patients within the primary analysis of ART failure at 12 months (**Figure 1**). More than half of patients in the original cohort lacked baseline CD4 results, follow-up CD4 results, or both, and were excluded from the analysis (n = 1,587). An additional 441 patients were able to be included in the secondary analysis of ART failure by 42 months, based upon having a follow-up CD4 available after 12 months (**Figure 1**). Patient characteristics are shown in **Tables 1** **–** **2**.

**Table 1:** Patient Characteristics (Categorical Variables).

	Full Study Cohort		Primary Analysis		Failure cases	
	(n = 2,510)		(n = 923)		(primary analysis)	
	n	%	n	%	n	%
Site						
HSA Jérémie	1,302	51.9%	453	49.1%	100/453	22.1%
HSM Jacmel	1,208	48.1%	470	50.9%	96/470	20.4%
Sex^a^						
Male	1,066	42.5%	382	41.4%	94/382	24.6%
Female	1,444	57.5%	541	58.6%	102/541	18.9%
Marital status						
Single	624	24.9%	227	24.6%	49/227	21.6%
Married/cohabitating	1,451	57.8%	539	58.4%	116/539	21.5%
Widowed/separated	343	13.7%	121	13.1%	21/121	17.4%
Unknown	92	3.7%	36	3.9%	10/36	27.8%
ART start year^b^						
2005–06	314	12.5%	135	14.6%	42/135	31.1%
2007–08	680	27.1%	296	32.1%	58/296	19.6%
2009–10	668	26.6%	225	24.4%	37/225	16.4%
2011–12	848	33.8%	267	28.9%	59/267	22.1%
Proximity of residence to site						
Same commune	861	34.3%	355	38.5%	77/355	21.7%
Adjacent commune	401	16.0%	124	13.4%	29/124	23.4%
Non-adjacent commune	1,205	48.0%	431	46.7%	88/431	20.4%
Missing	43	1.7%	13	1.4%	13-Feb	15.4%
ART starting regimen						
AZT-3TC-EFV	1,223	48.7%	464	50.3%	111/464	23.9%
AZT-3TC-NVP	725	28.9%	293	31.7%	56/293	19.1%
Other standard	509	20.3%	143	15.5%	21/143	14.7%
Non-standard	53	2.1%	23	2.5%	8/23	34.8%
Number of adherence assessments (months 1–6)^a^						
None	1,172	46.7%	340	36.8%	88/340	25.9%
One	156	6.2%	62	6.7%	10/62	16.1%
2 or more	1,182	47.1%	521	56.4%	98/521	18.8%
Any adherence assessment by clinician (months 1–6)						
No	1,846	73.5%	641	69.4%	138/641	21.5%
Yes	664	26.5%	282	30.6%	58/282	20.6%

AZT = zidovudine, 3TC = lamivudine, EFV = efavirenz, NVP = nevirapine.

Chi-square test p-values for difference in failure proportions by group, among patients included in the primary analysis:^ a^p<.05,

b p<0.01.

**Table 2:** Patient Characteristics (Continuous Variables).

	Full study cohort (n = 2,510)			Primary analysis (n = 923)			ART failure (n = 196)			No ART failure (n = 727)		
	n	mean	sd	n	mean	sd	n	mean	sd	n	Mean	sd
Age at ART start^a^	2510	38.4	11.4	923	38.7	10.9	196	37.3	11.3	727	39.0	10.8
Baseline CD4^1^	2120	208.0	182.9	923	207.8	183.1	196	267.1	266.7	727	191.8	149.2
Baseline BMI	1911	20.7	3.2	727	20.8	3.1	150	20.9	3.0	577	20.8	3.1
# days enrolled before ART start	2510	292.3	468.7	923	299.9	437.7	196	247.8	373.4	727	314.0	452.7

a p<.05 by Student t-test for difference in means between ART failures and non-failures, among patients included in the primary analysis.

1 p<0.001 by Wilcoxon rank sum test (non-parametric test) for equality of distributions between ART failures and non-failures, among patients included in the primary analysis.

Pharmacy-based adherence data were more readily available than self-reported adherence data, during the first 6 months on ART for all patients in the study (**Table 3**). While less than 5% of patients lacked pharmacy-based adherence measures, 40% of patients lacked self-reported adherence measures. Among patients enrolled on ART after the adherence assessment form was introduced in February 2008 for collecting self-reported measures, 17.5% lacked these measures during the first 6 months on ART. ART medications were dispensed on average for 40.8 days (IQR: 32–50 days), indicating that dispensing data were generally sufficiently frequent to calculate pharmacy-based adherence measures on a 3–6 month basis.

**Table 3:** Availability of Adherence Measures during First 6 Months on ART.

	Pharmacy-based measures			Self-reported measures	
	MPR	PDC	TVR	VAS	%NoMD
	n (%)	n (%)	n (%)	n (%)	n (%)
All patients (n = 2,510)					
available	2,458 (97.9%)	2,458 (97.9%)	2,242 (89.3%)	1,496 (59.6%)	1,505 (60.0%)
missing	52 (2.1%)	52 (2.1%)	268 (10.7%)	1,014 (40.4%)	1,005 (40.0%)
Patients enrolled on ART before ART adherence assessment form introduced (n = 696)					
available	645 (92.7%)	645 (92.7%)	589 (84.6%)	8 (1.1%)	8 (1.1%)
missing	51 (7.3%)	51 (7.3%)	107 (15.4%)	688 (98.9%)	688 (98.9%)
Patients enrolled on ART after ART adherence assessment form introduced (n = 1,814)					
available	1,813 (99.9%)	1,813 (99.9%)	1,653 (91.1%)	1,488 (82.0%)	1,497 (82.5%)
missing	1 (0.1%)	1 (0.1%)	161 (8.9%)	326 (18.0%)	317 (17.5%)

MPR = Medication possession ratio; PDC = Proportion of days covered; TVR =  Timely visit ratio; VAS = Visual analogue scale; %NoMD = Proportion of visits with no missed dose reported.

### Adherence Levels

**Figure 2:**
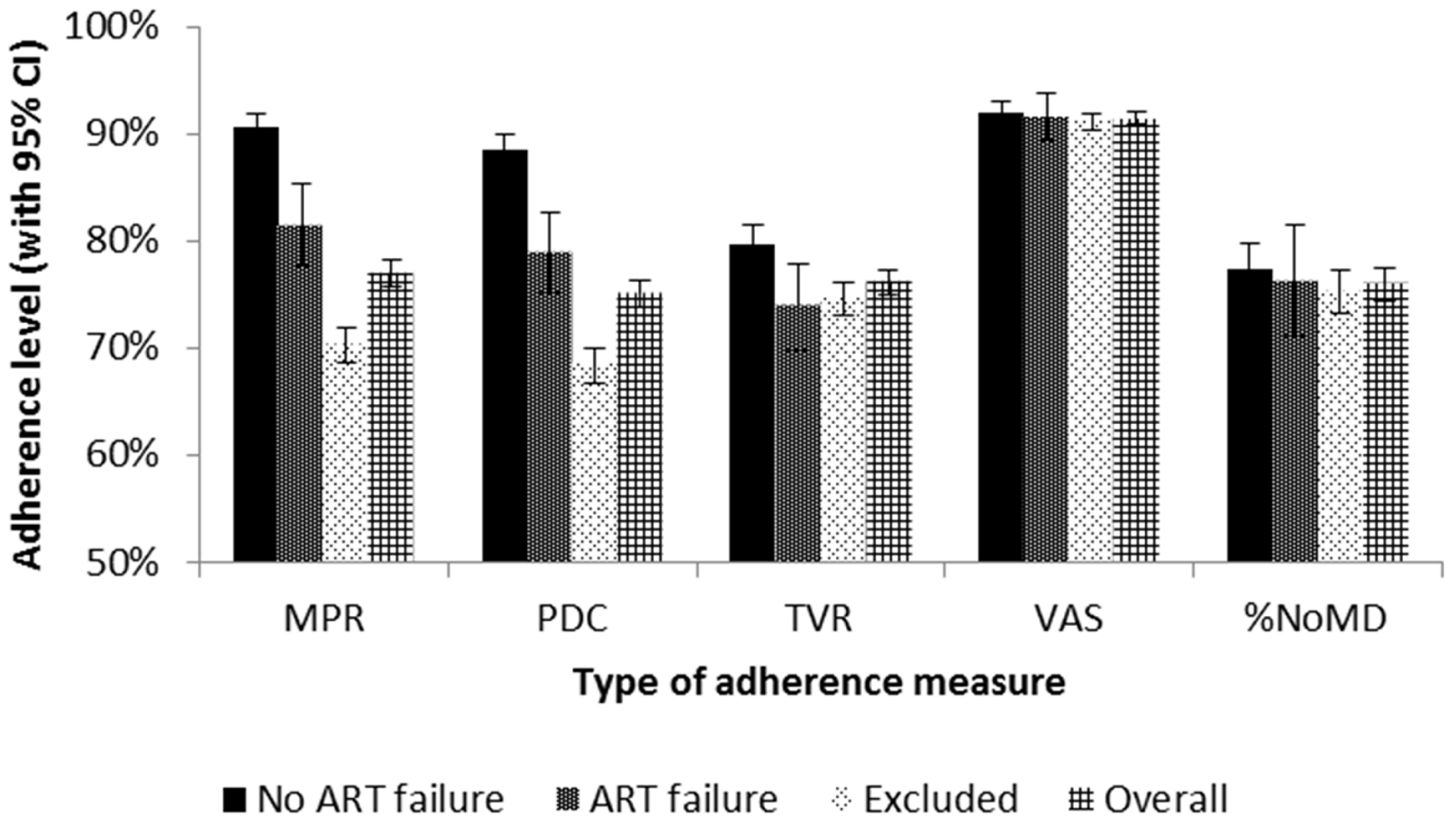
Baseline ART Adherence Levels (Months 1–6). *Pharmacy-based measures*. MPR = Medication possession ratio; sample size: n = 2,458. PDC = Proportion of days covered; sample size: n = 2,458. TVR =  Timely visit ratio; sample size: n = 2,242. *Self-reported adherence measures*. VAS = Visual analogue scale; sample size: n = 1,496. %NoMD = Proportion of visits with no missed dose reported; sample size: n = 1,505. *Comparison groups*. No ART failure (n = 727) and ART failure (n = 196) groups refer to patients in the primary analysis. Excluded group refers to patients excluded from the primary analysis (n = 1,587). Overall group refers to the full population of adult ART patients (n = 2,510).

In the overall cohort of 2,510 patients, mean adherence within the first 6 months was estimated to be highest based on the VAS measure (91.4%), followed by MPR (77.0%), TVR (76.1%), %NoMD (76.0%), and finally PDC (75.1%) (**Figure 2**). MPR and PDC were lower among the 1,587 patients who were excluded from the primary 12-month analysis than among the 923 patients who were included. Patients with no self-reported measures had markedly lower pharmacy-based adherence measures than those with self-reported measures available. Among those enrolled on ART after the introduction of the adherence assessment form and included in our primary analysis (n = 643), mean PDC was 88.7% among those with a self-reported measure but only 66.2% among those without one (p<0.001 by t-test). In other words, those who never provided self-reported adherence data tended to have poorer adherence as estimated by pharmacy data.

### Primary Analysis: ART failure by 12 months

Among the patients included in the primary analysis (n = 923), 196 (21.2%) met ART failure criteria by 12 months. Of the failure cases, 75% had a follow-up CD4 below the baseline value, 19% had a follow-up CD4 below 100 cells/µl, 5% met both of these CD4 criteria, and <1% had a new stage IV clinical diagnosis. ART failure was more typical among male patients (p = 0.04), those who started ART in 2005–06 (p<0.01), those with higher baseline CD4 level (p<0.001), and those with no adherence assessments conducted during the first 6 months on treatment (p = 0.03) (**Tables 1** **–** **2**).

The MPR and PDC measures differentiated cases of ART failure from non-failure, with non-overlapping 95% confidence intervals for mean measures in these two groups (**Figure 2**). Mean MPR and PDC levels were approximately nine percentage points lower among cases of failure vs. non-failure (p<0.001 for each). The mean TVR measure had slightly overlapping 95% confidence intervals in the ART failure vs. non-failure groups. In contrast, the self-reported measures did not differentiate the two groups and the 95% confidence intervals for the mean measures were largely overlapping.

In the first step of identifying a robust prediction model – that of selecting the best-performing adherence measure from among the five available measures – PDC demonstrated the best performance (AUC = 0.61; 95% CI: 0.56–0.66) (**Table 4**). In the second step, the stepwise covariate selection procedure conducted in the training dataset revealed several significant predictors of ART failure over and above PDC. These were male sex, higher baseline CD4 level, and lower duration of enrollment in HIV care prior to starting ART (“pre-ART duration”). The prediction model with both baseline adherence and these other patient characteristics performed better than models with only adherence or with only patient characteristics in the validation dataset (**Table 5**). The covariates for age, marital status, hospital site, proximity of residence, ART start year, initial ART regimen, baseline BMI, number of adherence assessments, and having any adherence assessment done by a clinician all failed to improve the AIC and were dropped from the prediction model. Predicted risk jumped with PDC<80%, with baseline CD4 values of >250 cells/µl, and with pre-ART duration of less than 160 days (data not shown).

**Table 4:** Performance of Baseline Adherence Measures as Predictors of ART Failure at 12 Months.

Measure	Functional form	n	AUC	95% CI	
MPR	continuous	899	0.60	(0.56,	0.65)
PDC	continuous	899	0.61	(0.56,	0.66)
TVR	continuous	875	0.56	(0.52,	0.61)
%NoMD	continuous	583	0.52	(0.46,	0.57)
VAS	continuous	582	0.50	(0.44,	0.56)

MPR: Medication possession ratio; PDC: Proportion of days covered; TVR: Timely visit ratio; VAS: Visual analogue scale; %NoMD: Proportion of visits with no missed dose reported; AUC: Area under the receiver operating curve; CI: confidence interval.

**Table 5:** Comparison of Prediction Models for ART Failure (12-month outcome).

	PDC only^1^				Patient characteristics only^1^				Both PDC and patient characteristics^1^				Simple model^2^			
	OR	95% CI		p-value	OR	95% CI		p-value	OR	95% CI		p-value	OR	95% CI		p-value
PDC	1.02	(1.01,	1.03)	0.002					1.01	(1.00,	1.02)	0.017	2.16	(1.27,	3.65)	0.004
Male					1.76	(1.11,	2.81)	0.017	2.12	(1.30,	3.47)	0.003	1.87	(1.15,	3.05)	0.011
Baseline CD4					1.03	(1.01,	1.04)	<0.001	1.02	(1.01,	1.04)	0.001	2.60	(1.54,	4.38)	0.001
Pre-ART duration					0.99	(0.99,	1.00)	0.018	0.99	(0.99,	1.00)	0.061	2.43	(1.54,	4.38)	<0.001
Constant	0.20	(0.15,	0.27)	<0.001	0.36	(0.18,	0.42)	<0.001	0.23	(0.15,	0.35)	<0.001	0.07	(0.06,	0.16)	<0.001
AIC	448.5				468.3				432.5				430.5			
		n	AUC			n	AUC			n	AUC			n	AUC	
Training sample		446	0.58			462	0.63			446	0.67			446	0.68	
Validation sample		453	0.64			461	0.64			453	0.69			453	0.69	

AIC: Akaike Information Criterion; PDC: Proportion of days covered; OR: Odds ratio; CI: Confidence interval.

1 Model uses untransformed continuous version of PDC, baseline CD4, and pre-ART duration;

2 Model uses binary version of PDC (<80%), baseline CD4 (> = 250), and pre-ART duration (<160).

A simplified prediction model using binary versions of each covariate performed as well as a model using continuous versions of covariates (**Table 5**). The risk score had possible values 0–32.5 and followed the equation:

*Risk Score*  = *7.7(pdc≤0.80)* +*9.6(cd4≥250)* +*8.9(duration≤160)* +*6.3(male sex)*

Grouping the risk score into tertiles for high, medium, and low risk also resulted in predictive discrimination in the validation sample, with an AUC of 0.63 (95% CI: 0.58–0.68). The proportion of patients experiencing ART failure by 12 months was 10.1% in the low risk group, 14.4% in the medium risk group, and 28.7% in the high risk group. The odds of ART failure at 12 months was 1.5 times higher for patients in the medium risk group (OR = 1.50; 95% CI: 0.66–3.40, p = 0.37) and 3.6 times higher for patients in the high risk group (OR = 3.58; 95% CI: 1.76–7.30, p<0.0001), compared to those in the low risk group (results not shown).

The implications of using each different risk grouping as a cut-off for a positive “risk test” are shown in **Table 6**. Using the low group as the cut-off would treat 100% patients as positive for the risk test and would be equivalent to no test. Using the medium group as the cut-off would identify approximately 75% of patients as positive for the risk test, while using the high group as the cut-off would classify approximately 50% of patients as positive for the risk test. The sensitivities, specificities, positive and negative predictive values for each risk test strategy, derived from the validation sample of the 12-month outcome study, are shown in **Table 6**. The implications of applying each risk test strategy to a hypothetical population of 1,000 patients, with and without resource constraints for actually targeting those with a positive risk test, are also shown in **Table 6**. In the absence of resource constraints, using no risk test would result in catching all eventual cases of ART failure, and would be the preferred strategy. However, when resource constraints are present and only half of all patients can be targeted, the fewest cases of ART failure would be missed when only those in the high risk grouping are defined as having a positive risk test and are targeted for services.

**Table 6:** Diagnostic “Risk Test” Characteristics under Different Strategies.

	Low + medium + high groups have positive “risk test” (no “risk test”)	Medium + high groups have positive “risk test”	High group has positive “risk test”
Test classification characteristics			
Sensitivity	100.0%	89.6%	70.8%
Specificity	0.0%	24.4%	53.7%
PPV	20.8%	23.8%	28.7%
NPV	NA	89.9%	87.5%
Correctly classified	20.8%	38.0%	57.3%
Hypothetical population of 1,000 with unlimited resources for targeting			
Total targeted	1000	785	514
Cases of failure among targeted	208	186	147
Cases of non-failure among targeted	792	599	367
Cases of failure missed	0	22	61
Hypothetical population of 1,000 but with resources to target only 500			
Total targeted	500	500	500
Cases of failure among targeted	104	119	143
Cases of non-failure among targeted	396	381	357
Cases of failure missed	104	89	65

PPV  =  positive predictive value; NPV  =  negative predictive value.

### Secondary Analysis: ART failure by 42 months

The 42-month outcome analysis supported the findings of the 12-month analysis. Among the 441 additional patients, baseline PDC was slightly higher than among patients in the 12-month study (89.4% vs. 86.5%, p = 0.01), but no other patient characteristics were significantly different. During the 42 months after ART initiation, 376 patients (28.9%) were observed to experience ART failure.

**Figure 3:**
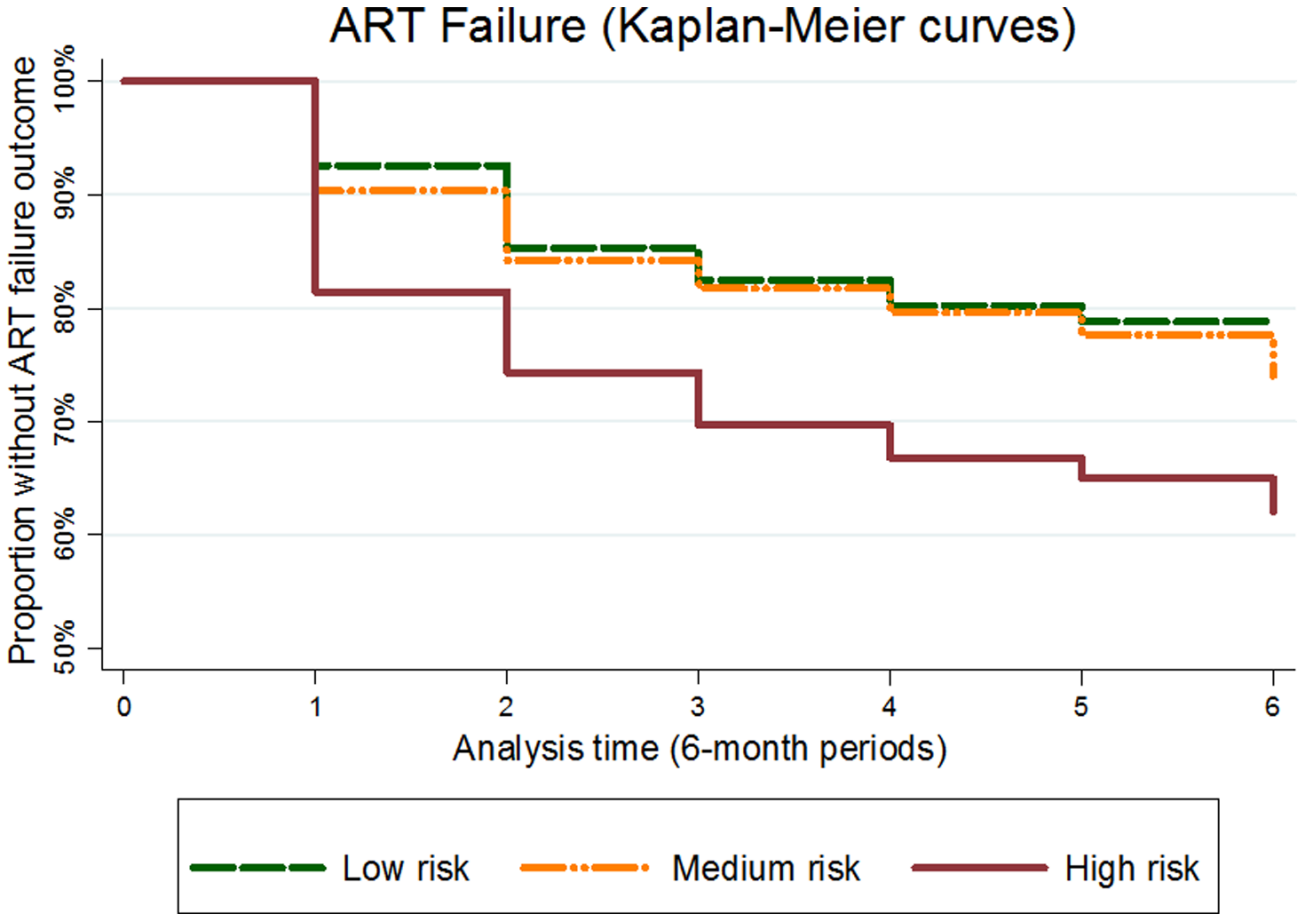
Kaplan Meier Curves for ART Failure by Risk Category (42-month outcome). Log rank test: p<0.001; CI: Confidence interval.

In the time-to-event analyses using standardized versions of the adherence measures, PDC was again the most predictive measure of ART failure. Kaplan-Meier survival curves stratified by the low, medium and high risk groupings showed a higher rate of ART failure in the high risk group compared to low and medium groups, and the curves suggest that this relationship between predicted risk and observed rate of failure holds even at time points up to 3.5 years after ART initiation (**Figure 3**). The difference in survival estimates among the groups was highly statistically significant (log rank p<0.0001).

### Sensitivity Analyses

Our sensitivity analyses also yielded consistent results. When patients without baseline CD4 values were included in the 12-month analysis, the number of included patients increased modestly from 923 to 1,005 patients. PDC continued to be the best-performing adherence measure. In an enriched prediction model, hospital site emerged as an additional predictive covariate beyond PDC, sex, baseline CD4 and pre-ART duration. Still, the original risk score demonstrated good discrimination beyond chance in the sensitivity analysis. Relaxing the requirement for a baseline CD4 increased the number of patients in the 42-month analysis from 1,302 to 1,511. As in the original analysis, stratification by risk grouping produced dramatic differences in the Kaplan-Meier survival curves (log-rank p<0.001).

## Discussion

Our study estimated that approximately one-fifth of ART patients met the WHO immunologic or clinical criteria for ART failure at 12 months. Pharmacy refill data, particularly the PDC adherence measure, had high informational value in predicting ART failure. In contrast, self-reported adherence measures were frequently missing and, when they were captured, they offered little value in discriminating patients who experienced ART failure. Male sex, higher CD4 level at baseline, and less time enrolled in care before initiating ART were also important predictors of ART failure. A risk score based on the baseline PDC measure together with these patient characteristics strongly discriminated patients at risk of ART failure. Whereas only 10.1% of patients in the lowest risk score grouping experienced ART failure, 28.7% in the highest risk score grouping did so. The risk grouping was robust in predicting failure up to 42 months following ART initiation, and also performed well in the context of missing baseline CD4 results.

Our results confirm PDC to be a viable proxy for adherence. The relative completeness of pharmacy-based data on medication regimen types, dispense dates, and amounts of medication dispensed, and the low likelihood that patients sought medications from sources outside their primary facility, all support use of iSanté pharmacy data to estimate ART adherence. Several additional patient characteristics enriched the prediction model by reflecting dimensions of adherence not captured in the PDC proxy measure. For example, patients with higher baseline CD4 may have been less motivated to consistently take the medication they had in their possession, based upon a better overall level of health. Patients with longer duration of pre-ART enrollment may have included those who are inherently likely to remain in care and comply with recommended care behaviors (since others may have dropped out before initiating ART), while patients with brief duration of pre-ART enrollment may have included patients with a mix of inherent tendencies toward compliance. Lower pre-ART duration has been identified as a risk factor for ART attrition in Haiti [30]. The higher risk of ART failure we found among males is consistent with evidence of elevated ART attrition risk among males in Haiti and elsewhere in the Caribbean region [31].

In a resource-limited setting like Haiti, our risk score has an important role to play in helping providers target patients for adherence support services before ART failure occurs. Such services could include in-depth counseling sessions with social workers or psychologists, adherence reminder phone calls or text messages, outreach visits by community health workers, or other supportive interventions. In the context of highly constrained human and material resources, it may not be possible to offer these interventions on a universal basis. This reality is underscored by the fact that nearly 20% of patients in our study population had no adherence assessment and counseling form completed. That these very patients tended to have lower adherence levels by pharmacy-based refill data, indicates that providers seemed to be missing patients with high levels of need. When resources are constrained, using the information about which patients are at higher risk of ART failure could help to decrease the number of eventual cases of ART failure which are missed. In a hypothetical cohort of 1,000 ART patients of whom only half can be reached with intensive adherence support services, targeting these services to high risk patients would result in missing 40% fewer of these critical cases.

Even if universal coverage with adherence support interventions is possible, knowing a patient's risk grouping could still help providers customize their communication messages according to risk grouping. For example, counseling for a patient at lower risk could focus on reinforcing positive behaviors. For those at higher risk, counseling could focus on identifying and overcoming barriers to adherence and could include referrals to other intensive adherence support services. This type of targeted communication strategy is identified as a best practice in HIV adherence support [32].

The first recommendation arising from our study is to use the validated risk scoring algorithm to program an automated provider alert within the iSanté electronic medical record system. The alert could be used in either an active or passive manner. For an iSanté alert to be successful, providers would need to receive training on how to interpret the alert and integrate its use within their care delivery processes. Well-designed electronic clinical alerts and reminders have been shown to improve quality of care and patient health outcomes [33], [34], [35], [36], [37].

The second recommendation arising from our study is to drop routine, universal documentation of self-reported adherence measures by providers. Leveraging automated adherence estimates derived from pharmacy refill data would allow for more efficient use of health worker and patient time, eliminating the need for providers to collect and record self-reported adherence data for patients with already-strong adherence by pharmacy data. Pharmacy personnel, who were responsible for completing about half of the adherence assessments for patients in our study, could shift their attention toward assuring strong data quality in the pharmacy dispense and refill data, as well as toward following up with patients who are late for pharmacy refills. Having providers ask patients about adherence levels and barriers could have value as a cue to favorable adherence behaviors among patients; so it is important to note that the recommendation is not to abandon these conversations but rather to drop universal data collection of the self-reported adherence measures.

### Strengths and Limitations

A strength of our study is the exploration of several types of adherence measures in order to identify the measure which performed best in predicting ART failure. Another strength is that in evaluating various prediction models, we looked for a balance between the simplicity of the model and the robustness of its statistical performance. For an automated alert to be effective in Haiti, it is important that it be well-understood and trusted by Haitian providers. Our four-factor model, using binary thresholds for PDC, baseline CD4, and pre-ART duration, can be easily understood as representing a common “profile” of patients at high risk of ART failure. The risk groupings with low, medium, and high categories are also intuitive. A final strength of our study is the use of a randomly split sample in developing and validating the risk score. The strong performance of the risk score and risk grouping within the validation sample speaks favorably to the generalizability of our results to other patient populations and at other sites.

A key limitation of our study is the fact that many patient records lacked either baseline or follow-up CD4 values, meaning they lacked data on the ART failure outcome. Both our secondary and our sensitivity analyses supported the primary findings when including patients with follow-up CD4 results only available after 12 months and patients with no baseline CD4 results. Further, the distribution of sex and the average levels of baseline CD4 and pre-ART duration did not differ between patients included vs. excluded from the primary analysis. This gives modest evidence for the generalizability of our findings to patients who were excluded from the primary analysis.

A second concern related to generalizability of our findings is that our study examined ART patients from only two facilities, representing approximately 6% of all ART patients within the iSanté data system. ART patients at HSA and HSM may be different from patients in other sites. Other iSanté sites might not share the relatively high completeness of pharmacy data we found in our cohort. Other sites may also have implemented the self-reported adherence assessments in a different manner, rendering the information more useful.

A third limitation of our study is that AUC values of above 0.70 are typically considered desirable for screening and diagnostic tests. An algorithm for ART failure risk developed for several large-scale electronic medical record systems in Boston demonstrated an AUC of 0.78, indicating strong discrimination [38]. ART adherence measures in South Africa had AUC values of 0.67–0.74 in predicting virologic failure at 6 or 12 months [39]. Our study found slightly lower AUC values, indicating weaker ability to discriminate ART failures from non-failures. Still, our risk score grouping was notably better than chance in predicting ART failure and would be useful in targeting patients, particularly since no harmful procedures would be triggered by false positive results of the risk test.

HIV viral load testing, the “gold standard” measure of ART failure, is not widely available in Haiti. There can be substantial misclassification of ART failure status when only immunologic and clinical status is known and no confirmatory viral load testing is available [40], [41], [42], [43]. Still, the WHO immunologic and clinical criteria for ART failure represent the available information upon which clinicians in Haiti must act. If viral load becomes more available in the future in Haiti, it would be possible to repeat the study methodology to validate a simple and robust prediction model for this “gold standard” outcome.

Several important patient-level factors which might be important predictors of ART adherence and ART failure — such as employment status, socioeconomic status, food security, level of education, mental health status, drug and alcohol use, pill burden, and concurrent treatments with potential for drug interactions and additional side effects — were also not available within iSanté or were not available for the purposes of the study. Data on timing and frequency of ART stock-outs at the health facilities were also unavailable. Whether patients were infected with drug-resistant strains of HIV before initiating ART was also unknown, due to the lack of routine drug susceptibility testing. In the only published study on ART drug resistance in Haiti, 40% of urban adolescents were found to have drug resistance after 12 months on ART [44], indicating the potential for concerning circulation of drug-resistant strains of HIV in the population.

## Conclusions

There is a critical need to support high ART adherence among patients placed on first-line ART regimens in resource-limited settings, to avoid cases of ART failure. Electronic medical record systems like iSanté can return relevant, actionable information to clinicians so that they can identify patients with problematic adherence and high risk of ART failure. Our study results support the development of an automated alert, leveraging iSanté pharmacy refill data as well as other patient characteristics, to predict risk of ART failure before it occurs. Such an alert could help in targeting patients with greatest need for in-depth ART adherence counseling and other supportive services. It would also help providers customize counseling messages according to the patient's level of risk. In the context of limited resources, there is high informational value in an alert which can improve targeting of patients in these ways.
